# Editorial: Community series in Th2-associated immunity in the pathogenesis of systemic lupus erythematosus and rheumatoid arthritis, volume II

**DOI:** 10.3389/fimmu.2025.1633681

**Published:** 2025-06-03

**Authors:** Quanren Pan, Andrew F. Walls, Qingjun Pan

**Affiliations:** ^1^ Department of Clinical Laboratory, State Key Laboratory of Respiratory Disease, The First Affiliated Hospital of Guangzhou Medical University, Guangzhou, China; ^2^ Guangdong Provincial Key Laboratory of Autophagy and Major Chronic Non-Communicable Diseases, Clinical Research and Experimental Center, Department of Nephrology, Affiliated Hospital of Guangdong Medical University, Zhanjiang, China; ^3^ School of Clinical and Experimental Sciences, Faculty of Medicine, University of Southampton, Southampton, United Kingdom

**Keywords:** autoimmune diseases, systemic lupus erythematosus, rheumatoid arthritis, T helper cells, extracellular vesicles, long non-coding RNAs, machine learning, gut microbiota

## 1 CD4^+^ T helper (Th) cells as central coordinators in autoimmune pathogenesis

CD4^+^ T helper (Th) cells orchestrate immune responses through cytokine-mediated signaling and intercellular communication. Th2-polarized immunity plays dual regulatory roles in autoimmune pathogenesis, mediated not only by canonical cytokines (IL-4, IL-5, IL-13) but also by extracellular vesicle trafficking, transcriptional regulators (Gata3/Batf/Irf4 axis), and bidirectional crosstalk with innate immune cells (1–3). These mechanisms amplify humoral responses and perpetuate tissue inflammation in systemic lupus erythematosus (SLE) and rheumatoid arthritis (RA). This review synthesizes advances in Th2-linked pathways, including novel biomarkers, therapeutic strategies, and disease mechanisms. Five studies address breakthroughs in SLE-pulmonary arterial hypertension (PAH) pathogenesis, lupus nephritis (LN) prognostication, RA diagnostics, and atherosclerosis management, offering actionable insights for precision medicine.

## 2 Telitacicept in SLE: balancing dose-dependent efficacy and safety

SLE pathogenesis involves B-cell hyperactivity driven by B-lymphocyte stimulator (BLyS) and a proliferation-inducing ligand (APRIL) (4). Telitacicept, a dual BLyS/APRIL inhibitor, received conditional approval for SLE in China in 2021 (4). To evaluate its efficacy and safety, Gao et al. conducted a meta-analysis of three randomized controlled trials (RCTs; *n* = 606 SLE patients). All doses (80 mg, 160 mg, 240 mg) significantly improved SLE Responder Index 4 (SRI4; RRs: 2.20–2.44), SELENA-SLEDAI (RRs: 1.63–1.73), and Physician Global Assessment (PGA; RRs: 1.24–1.39) scores versus controls (*P* ≤ 0.002). Notably, the 160 mg dose uniquely enhanced British Isles Lupus Assessment Group (BILAG) scores (RR = 1.11, *P* = 0.03) but increased adverse event risk (RR = 1.10, *P* = 0.007). These findings suggest 160 mg as a critical threshold: lower doses for safety-focused regimens and 160 mg for refractory cases. However, phase IV trials are urgently needed to validate protocols balancing efficacy and safety in real-world settings.

## 3 EVs as biomarkers and mediators in SLE-PAH

Pulmonary arterial hypertension (PAH), a life-threatening SLE complication, drives progressive right heart failure (5). Endothelial extracellular vesicles (eEVs) exacerbate PAH via procoagulant activity and pulmonary embolism (6). In a recent study, Ding et al. observed elevated circulating EV subpopulations—leukocyte-derived EVs (LEVs) and red blood cell-derived EVs (REVs)—in 18 SLE-PAH patients compared to non-PAH SLE controls. LEVs and REVs correlated with pulmonary arterial systolic pressure, right ventricular diameter, and hypercoagulation (Annexin V+ subtypes showed the strongest associations). Multivariate analysis identified LEVs, REVs, anti-nRNP antibodies, and serositis as independent PAH risk factors. Receiver operating characteristic (ROC) analysis supported EVs as predictive biomarkers. Despite limitations (small sample size, lack of longitudinal data), this study highlights EVs as promising tools for diagnosing thrombotic complications and monitoring SLE-PAH severity.

## 4 Exosomal lncRNAs: non-invasive biomarkers for RA

RA is characterized by chronic joint inflammation and bone erosion, though its pathogenesis remains incompletely understood (7). To address diagnostic challenges, Wu et al. identified serum exosomal long non-coding RNAs (lncRNAs)—TCONS_I2_00013502 (upregulated) and ENST00000363624 (downregulated)—as RA biomarkers. Whole-transcriptome sequencing of exosomes from 120 RA patients (stratified by disease activity and medication) confirmed consistent dysregulation. ROC analysis demonstrated high diagnostic accuracy (AUC > 0.85). Functional enrichment analysis linked these lncRNAs to immune regulation, though mechanistic pathways require further exploration. While promising, both validation in diverse cohorts and mechanistic studies are needed to establish their clinical utility.

## 5 Machine learning-driven prognostic modeling in lupus nephritis

The 2003 International Society of Nephrology/Renal Pathology Society (ISN/RPS) classification stratifies LN into six classes, with class IV ± V associated with poor renal outcomes (8, 9). Notably, Wang et al. analyzed 313 class IV ± V LN patients and found comparable survival outcomes between subgroups despite histopathologic differences (higher chronicity index in class IV, *P* < 0.001). Using a random survival forest (RSF) model, they integrated seven predictors—estimated glomerular filtration rate (eGFR), chronicity index, age, basophil percentage, red blood cell count, mean arterial pressure, and uric acid—to forecast renal endpoints (eGFR decline >50%, end-stage renal disease, or death). The model demonstrated strong discrimination (C-index = 0.771) and calibration (integrated Brier score = 0.144), validated by integrated discrimination improvement (IDI) and net reclassification improvement (NRI) analyses. This challenges existing prognostic hierarchies and advocates for unified management strategies, pending external validation.

## 6 SLE, atherosclerosis, and therapeutic horizons

SLE elevates cardiovascular disease (CVD) risk, with atherosclerosis contributing significantly to mortality (10). A comprehensive review by Pan et al. implicates immune dysregulation, gut microbiota alterations, and metabolic disturbances in accelerating atherosclerosis in SLE. SLE exacerbates atherosclerosis via hyperactivated immune cells, autoantibodies, and chronic inflammation. Despite current therapies (statins, anti-inflammatory agents, T/B-cell inhibitors), robust validation in SLE-specific contexts remains lacking. The authors propose investigating gut microbial strains and metabolites as novel biomarkers or therapeutic targets to mitigate cardiovascular mortality in SLE.

## 7 Conclusion

This Research Topic bridges translational gaps in Th2-associated immunity, from telitacicept dose optimization to EV-mediated thrombosis and lncRNA-based diagnostics. Collectively, these studies advance precision medicine in SLE and RA through biomarker-driven stratification, prognostic modeling, and mechanism-targeted therapies. Future efforts must prioritize large-scale validation, mechanistic exploration, and interdisciplinary collaboration to translate these insights into clinical practice, ultimately improving outcomes for autoimmune disease patients.
